# Complex systems and the technology of variability analysis

**DOI:** 10.1186/cc2948

**Published:** 2004-09-22

**Authors:** Andrew JE Seely, Peter T Macklem

**Affiliations:** 1 Assistant Professor, Thoracic Surgery and Critical Care Medicine, University of Ottawa, Ottawa, Ontario, Canada; 2 Professor Emeritus, Respiratory Medicine, McGill University, Montreal, Quebec, Canada

**Keywords:** complex systems, critical illness, entropy, therapeutic monitoring, variability

## Abstract

Characteristic patterns of variation over time, namely rhythms, represent a defining feature of complex systems, one that is synonymous with life. Despite the intrinsic dynamic, interdependent and nonlinear relationships of their parts, complex biological systems exhibit robust systemic stability. Applied to critical care, it is the systemic properties of the host response to a physiological insult that manifest as health or illness and determine outcome in our patients. Variability analysis provides a novel technology with which to evaluate the overall properties of a complex system. This review highlights the means by which we scientifically measure variation, including analyses of overall variation (time domain analysis, frequency distribution, spectral power), frequency contribution (spectral analysis), scale invariant (fractal) behaviour (detrended fluctuation and power law analysis) and regularity (approximate and multiscale entropy). Each technique is presented with a definition, interpretation, clinical application, advantages, limitations and summary of its calculation. The ubiquitous association between altered variability and illness is highlighted, followed by an analysis of how variability analysis may significantly improve prognostication of severity of illness and guide therapeutic intervention in critically ill patients.

## Introduction

Biological systems are complex systems; specifically, they are systems that are spatially and temporally complex, built from a dynamic web of interconnected feedback loops marked by interdependence, pleiotropy and redundancy. Complex systems have properties that cannot wholly be understood by understanding the parts of the system [1]. The properties of the system are distinct from the properties of the parts, and they depend on the integrity of the whole; the systemic properties vanish when the system breaks apart, whereas the properties of the parts are maintained. Illness, which presents with varying severity, stability and duration, represents a systemic functional alteration in the human organism. Although illness may occasionally be due to a specific singular deficit (e.g. cystic fibrosis), this discussion relates to illnesses characterized by systemic changes that are secondary to multiple deficits, which differ from patient to patient, with varied temporal courses, diverse contributing events and heterogeneous genetic contributions. However, all factors contribute to a physiological alteration that is recognizable as a systemic illness. Multiple organ dysfunction syndrome represents the ultimate multisystem illness, really representing a common end-stage pathway of inflammation, infection, dysfunctional host response and organ failure in critically ill patients, and frequently leading to death [2]. Although multiple organ dysfunction syndrome provides a useful starting point for discussion regarding complex systems and variability analysis [3], the application of variability analysis to other disease states is readily apparent and exciting.

Life is composed of and characterized by rhythms. Abnormal rhythms are associated with illness and can even be involved in its pathogenesis; they have been termed 'dynamical diseases' [4]. Measuring the absolute value of a clinical parameter such as heart rate yields highly significant, clinically useful information. However, evaluating heart rate variability (HRV) provides additionally useful clinical information, which is, in fact, more valuable than heart rate alone, particularly when heart rate is within normal limits. Indeed, as is demonstrated below, there is nothing 'static' about homeostasis. Akin to the concept of homeorrhesis (dynamic stability) introduced by CH Waddington, homeokinesis describes 'the ability of an organism functioning in a variable external environment to maintain a highly organized internal environment, fluctuating within acceptable limits by dissipating energy in a far from equilibrium state' [5].

Clinicians have long recognized that alterations in physiological rhythms are associated with disease. The human eye is an excellent pattern recognition device, which is capable of complex interpretation of ECGs and electroencephalograms (EEGs) [6], and physicians make use of this skill on a daily basis. However, more sophisticated analysis of variability provides a measure of the integrity of the underlying system that produces the dynamics. As the spatial and temporal organization of a complex system define its very nature, changes in the patterns of interconnection (connectivity) and patterns of variation over time (variability) contain valuable information about the state of the overall system, representing an important means with which to prognosticate and treat our patients [3]. As clinicians, our goal is to make use of this observation in order to improve patient care. This technology of variability analysis is particularly valuable in the intensive care unit (ICU), where patients are critically ill and numerous parameters are routinely measured continuously. The intensivist is poised to marshal the science of variability analysis, becoming a 'dynamicist' [6], to measure and characterize the variability of physiological signals in an attempt to understand the information locked in the 'homeokinetic code' [7], and thus contribute to a breakthrough in our ability to treat critically ill patients.

The focus of this review and analysis is the measurement and characterization of variability, a science that has undergone considerable growth in the past two decades. The development of mathematical techniques with a theoretical basis in chaos theory and nonlinear dynamics has provided us with greater ability to discern meaningful distinctions between biological signals from clinically distinct groups of patients. The science of variability analysis has developed from a close collaboration between mathematicians, physicists and clinicians. As such, the techniques for measuring variability sometimes represent a bewildering morass of equations and terminology. Each technique represents a unique and distinct means of characterizing a series of data in time. The principal objectives of this review are as follows: to present a concise summary, including definition, interpretation, advantages, limitations and calculation of the principal techniques for performing variability analysis; to discuss the interpretation and application of this technology; and to propose how this information may improve patient care. Although the majority of the discussion relates to the analysis of HRV because is it readily and accurately measured on an ECG, the techniques are applicable to any biological time signal. Two tables are included to facilitate review of the techniques for characterizing variability (Table 1) and the evidence for altered variability in illness (Table 2).

## Science of variability analysis

### Sampling

The analysis of patterns of change over time or variability is performed on a series of data collected continuously or semicontinuously over time. For example, a heart rate tracing may be converted to a time series of intervals between consecutive heart beats (measured as R–R' intervals on an ECG). The same may be done with inter-breath intervals, albeit not as easily. When there is no intrinsic rhythm such as a heart or respiratory rate, sampling a signal occurs in discrete time intervals (e.g. serum concentrations of a hormone measured every few minutes). In order to reconstruct the underlying signal without error, one must respect the Nyquist Theorem, which states that the sampling frequency must be at least twice the highest frequency of the signal being sampled.

### Stationarity

Stationarity defines a limitation in techniques designed to characterize variability. It requires that statistical properties such as mean and standard deviation of the signal remain the same throughout the period of recording, regardless of measurement epoch. Stationarity does not preclude variability, but it provides boundaries for variability such that variability does not change with time or duration of measurement. If this requirement is not met, as is the case with most if not all biological signals when physiological and/or pathophysiological conditions change, then the impact of trends with change on the mean of the data set must be considered in the interpretation of the variability analysis. The relative importance of stationarity to individual techniques of variability analysis is addressed below.

### Artifact

Variability analysis should be performed on data that are free from artifact, with a minimal noise:signal ratio. Noise is measurement error, or imprecision secondary to measurement technology. Often present in patient monitoring, artifact must be removed, often by visual inspection of the raw data. For example, in the evaluation of HRV the presence of premature atrial and/or ventricular beats require that the data be removed, and appropriate interpolation be performed without compromising the integrity of the variability analysis. Several techniques, such as a Poincaré Plot of the difference between consecutive data points, have been developed to facilitate automated identification and removal of artifact [8-10]. Different techniques are more or less sensitive to artifact, which again is addressed below.

### Standardized technique

Various factors alter variability measurement. For example, standing or head-up tilt (increased sympathetic activity) and deep breathing (increased respiratory rate induced HRV) will alter HRV indices in healthy individuals. With deference to Heisenberg, experimental design should take into account that the process of measurement may alter the intrinsic variation. An important component of standardized technique is the duration of measurement for analysis. For example, indices of HRV may be calculated following a duration of 15 min or 24 hours. In general terms, it is inappropriate to compare variability analysis from widely disparate durations of measurements [11]. More specifically, the impact of duration of measurement varies in relation to individual analysis technique, and is discussed below.

## Time domain analysis

### Definition

Time series analysis represents the simplest means of evaluating variability, identifying measures of variation over time such as standard deviation and range. For example, quantitative time series analysis is performed on heart rate by evaluating a series of intervals between consecutive normal sinus QRS complexes (normal–normal, or NN or RR' interval) on an ECG over time. In addition, a visual representation of data collected as a time series may be obtained by plotting a frequency distribution, plotting the number of occurrences of values in selected ranges of values or bins.

### Calculation

Mathematically, standard deviation is equal to the square root of variance; and variance is equal to the sum of the squares of difference from the mean, divided by the number of degrees of freedom. Evaluating HRV, the standard deviation of a series of NN intervals (SDNN) represents a coarse quantification of overall variability. As a measure of global variation, standard deviation is altered by the duration of measurement; longer series will have greater SDNN. Thus, SDNN can be calculated for short periods between 30 s and 5 min and used as a measure of short-term variability, or calculated for long periods (24 hours) as a measure of long-term variation [12]. Because it is inappropriate to compare SDNNs from recordings of different duration, standardized duration of recording has also been suggested [11].

Various permutations of measurement of standard deviation, in an effort to isolate short-term, high frequency fluctuations from longer term variation, are possible. For example, SDANN (standard deviation of the average NN interval calculated over 5-min intervals within the entire period of recording) is a measure of longer term variation because the beat-to-beat variation is removed by the averaging process. In contrast, the following variables were devised as a measure of short-term variation: RMSSD (square root of the mean squared differences of consecutive NN intervals), NN50 (number of pairs of adjacent NN intervals differing by more than 50 ms), and pNN50 (proportion of NN intervals differing by more than 50 ms = NN50 divided by total number of NN intervals). These measures of high frequency variation are interrelated; however, RMSSD has been recommended because of superior statistical properties [11]. The conventional 50 ms used in the NN50 and pNN50 measurements represents an arbitrary cutoff, and is only one member of a general pNNx family of statistics; in fact, a threshold of 20 ms may demonstrate superior discrimination between physiological and pathological HRV [13].

In order to characterize a frequency distribution, it may be fitted to a normal distribution, or rather a log-normal distribution – one in which the log of the variable in question is normally distributed. The skewness or degree of symmetry may be calculated, with positive and negative values indicating distributions with a right-sided tail and a left-sided tail, respectively. Kurtosis may also be calculated to identify the peakedness of the distribution; positive kurtosis (leptokurtic) indicates a sharp peak with long tails, and negative kurtosis (platykurtic) indicates a flatter distribution.

### Interpretation and clinical application

Time domain analysis involves the statistical evaluation of data expressed as a series in time. Clinical evaluation of time domain measures of HRV have been extensive, using overall standard deviation (SDNN) to measure global variation, standard deviation of 5-min averages (SDANN) to evaluate long-term variation, and the square root of mean squared differences of consecutive NN intervals (RMSSD) to measure short-term variation. An abridged review of an extensive literature suggests that diminished overall HRV measured with time domain analysis portends poorer prognosis and/or increased mortality risk in patients with coronary artery disease [14,15], dilated cardiomyopathy [16], congestive heart failure [17,18] and postinfarction patients [19-23], in addition to elderly patients [24]. Time domain HRV analysis has been used to compare ß-blocker therapies postinfarction [25], to evaluate percutaneous coronary interventions [26,27], to predict arrhythmias [28] and to select patients for specific antiarrhythmic therapies [29], which are a few examples of a vast body of literature that is well reviewed elsewhere [30,31].

Time series of parameters derived from biological systems are known to follow log-normal frequency distributions, and deviations from the log-normal distribution have been proposed to offer a means with which to characterize illness [32]. For example, in paediatric ICU patients with organ dysfunction, HRV evaluated using a frequency distribution (plotting frequency of occurrence of differences from the mean) revealed a reduction in HRV and a shift in the frequency distribution to the left with increasing organ failure; these changes improved in surviving patients and were refractory in nonsurvivors [33]. The authors utilized a technique that was initially described in the evaluation of airway impedance variability, demonstrating increased variability in asthma patients characterized by altered frequency distribution [5].

### Advantages and limitations

Statistical measures of variability are easy to compute and provide valuable prognostic information about patients. Frequency distributions also offer an accurate, visual representation of the data, although the analysis may be sensitive to the arbitrary number of bins chosen to represent the data. Time domain measures are susceptible to bias secondary to nonstationary signals. A potential confounding factor in characterizing variability with standard deviation is the increase in baseline heart rate that may accompany diminished HRV indices. The clinical significance of this distinction is unclear, because the prognostic significance of altered SDNN or SDANN remains clinically useful. A more condemning limitation of time domain measures is that they do not reliably distinguish between distinct biological signals. There are many potential examples of data series with identical means and standard deviations but with very different underlying rhythms [34]. Therefore, additional, more sophisticated methods of variability analysis are necessary to characterize and differentiate physiological signals. It is nonetheless encouraging that, using rather crude statistical measures of variability, it is possible to derive clinically useful information.

## Frequency domain analysis

### Definition

Physiological data collected as a series in time, as with any time series, may be considered a sum of sinusoidal oscillations with distinct frequencies. Conversion from a time domain to frequency domain analysis is made possible with a mathematical transformation developed almost two centuries ago (1807) by the French mathematician Jean-Babtiste-Joseph Fourier (1768–1830). Other transforms exist (e.g. wavelet, Hilbert), but Fourier was first and his transformation is used most commonly. The amplitude of each sine and cosine wave determines its contribution to the biological signal; frequency domain analysis displays the contributions of each sine wave as a function of its frequency. Facilitated by computerized data harvest and computation, the result of converting data from time series to frequency analysis is termed spectral analysis because it provides an evaluation of the power (amplitude) of the contributing frequencies to the underlying signal.

### Calculation

The clinician should note that the power spectrum is simply a different representation of the same time series data, and the transformation may be made from time to frequency and back again. It is not necessary for the clinician to know how to perform power spectral density analysis using the fast Fourier transformation because computers can do so quickly and reliably, calculating a weighted sum of sinusoidal waves, with different amplitudes and frequencies. This provides an analysis of the relative contributions of different frequencies to the overall variation in a particular data series. Interpretation of the analysis must factor in the assumptions inherent to this calculation, namely stationarity and periodicity. Note that the square of the contribution of each frequency is the power of that frequency to the total spectrum, and the total power of spectral analysis (area under the curve of the power spectrum) is equal to the variance described above (they are different representations of the same measure) [11]. The fast Fourier transform or analysis (see Appendix 1) represents a nonparametric calculation because it provides an evaluation of the contribution of all frequencies, not discrete or preselected frequencies.

### Interpretation and clinical application

Spectral analysis of heart rate was first performed by Sayers [35]. It was subsequently used to document the contributions of the sympathetic, parasympathetic and renin–angiotensin systems to the heart rate power spectrum, which introduced frequency domain analysis as a sensitive, quantitative and noninvasive means for evaluating the integrity of cardiovascular control systems [36]. Spectral analysis has been utilized to evaluate and quantify cardiovascular and electroencephalographic variability in numerous disease states, and is perceived as an important tool in clinical medicine [37].

The power spectral density function or power spectrum provides a characteristic representation of the contributing frequencies to an underlying signal. By identifying and measuring the area of distinct peaks on the power spectrum, it is possible to derive quantitative connotation to facilitate comparison between individuals and groups. In 2–5 min recordings, spectral analysis reveals three principal peaks, identified by convention with the following ranges: very low frequency (VLF; frequency = 0.04 Hz [cycles/s], cycle length >25 s), low frequency (LF; frequency 0.04–0.15 Hz, cycle length >6 s) and high frequency (HF; frequency 0.15–0.4 Hz, cycle length 2.5–6 s). In 24 hour recordings VLF is further subdivided into VLF (frequency 0.003–0.04 Hz) and ultralow frequency (ULF; frequency = 0.003 Hz, cycle length >5 hours) [11]. Correlations between time and frequency measures have also been demonstrated, for example in healthy newborns [38] and in cardiac patients following myocardial infarction [39].

Numerous factors in health and disease have an impact on the amplitude and area of each peak (or frequency range) on the HRV power spectrum. Akselrod and coworkers [36] first demonstrated the contributions of sympathetic and parasympathetic nervous activity and the renin–angiotensin system to frequency specific alterations in the HRV power spectrum in dogs. Several authors have evaluated and reviewed the relationship between the autonomic nervous system and spectral analysis of HRV [40-44]. Although autonomic regulation is clearly a significant regulator of the HRV power spectrum, evidence demonstrates a lack of concordance with direct evaluation of sympathetic tone, for example in patients with heart failure [45], and reviews increasingly conclude that HRV is generated by multiple physiological factors, not just autonomic tone [46,47].

In interpreting the significance of the HRV power spectrum, investigators initially focused on peaks because of a presumed relationship with a single cardiovascular control mechanism leading to rhythmic oscillations; however, others documented nonrhythmic (no peak) fluctuations in both heart rate and blood pressure variability, indicating the need to analyze broadband power [48]. Thus, the calculation of HF, LF, VLF and ULF using the ranges listed above serve to facilitate data reporting and comparison, but they are nonetheless arbitrary ranges with diverse physiological input. A recent review of HRV [47] documented the evidence that ULF reflects changes secondary to the circadian rhythm, VLF is affected by temperature regulation and humoral systems, LF is sensitive to cardiac sympathetic and parasympathetic nerve activity, and HF is synchronized to respiratory rhythms, primarily related to vagal innervation.

What does spectral analysis of HRV tell us about our patients? Despite nonspecific pathophysiological mechanisms, there is ample evidence that the frequency contributions to HRV are altered in illness states, and that the degree of alteration correlates with illness severity. It is illustrative that alterations in the spectral HRV analysis related to illness severity have been demonstrated from hypovolaemia [49] to heart failure [50-52], from hypertension [53,54] to coronary artery disease [55,56], and from angina [57] to myocardial infarction [58], in addition to chronic renal failure [59], autonomic neuropathy secondary to diabetes mellitus [60], depth of anaesthesia [61] and more. Spectral analysis of HRV has been applied in the ICU. For example, using spectral HRV and blood pressure variability analyses in consecutive patients admitted to an ICU, increasing total and LF HRV power were associated with recovery and survival, whereas progressive decreases in HRV were associated with deterioration and death [62]. In separate investigations involving patients in the emergency room [63] or admitted to an ICU after 48 hours [64], decreased total, LF and LF/HF HRV was not only present in patients with sepsis but also correlated with subsequent illness severity, organ dysfunction and mortality. Several reviews discuss the application of HRV spectral analysis to the critically ill patient [65-68]. Thus, alterations in spectral analysis correlate with severity of illness, a finding consistently reported in cardiac and noncardiac illness states, providing the clinician with a means with which to gauge prognosis and determine efficacy of intervention.

### Advantages and limitations

In order to derive a valid and meaningful analysis using a fast Fourier transform and frequency domain analysis, the assumptions of stationarity and periodicity must be fulfilled. The signal must be periodic, namely it is a signal that is comprised of oscillations repeating in time, with positive and negative alterations [69]. In the interpretation of experimental data, periodic behaviour may or may not exist when evaluating alterations in spectral power in response to intervention. The assumption of stationarity may also be violated with prolonged signal recording. Changes in posture, level of activity and sleep patterns will alter the LF and HF components of spectral analysis [70]. Spectral analysis is more sensitive to the presence of artifact and/or ectopy than time domain statistical methods. In addition, given that different types of Holter monitors may yield altered LF signals [71], it is essential to ensure that the sampling frequency of the monitor used to read QRS complexes does not contribute to error in the variability analysis [11,72]. Thus, the performance and interpretation of spectral analysis must incorporate these limitations. Recommendations based upon the stationarity assumption include the following [11]: short-term and long-term spectral analyses must be distinguished; long-term spectral analyses are felt to represent averages of the alterations present in shorter term recordings and may hide information; traditional statistical tests should be used to test for stationarity when performing spectral analysis; and physiological mechanisms that are known to influence HRV throughout the period of recording must be controlled.

### Time spectrum analysis

Another means to address the stationarity assumption inherent in the Fourier transform is to evaluate the power spectral density function for short periods of time when stationarity is assumed to be present, and subsequently follow the evolution of the power spectrum over time [73]. This combined time varying spectral analysis allows the continuous evaluation of change in variability over time. One can use sequential spectral approach [74], Wavelet analysis [75], the Wigner-Ville technique or Walsh transforms, all of which provide an analysis of frequency alteration over time, which is useful in clinical applications [37]. For example, time frequency analysis has demonstrated increased LF HRV power during waking hours (considered primarily a marker of sympathetic tone) and increased HF HRV during sleep (thought to be related to respiratory fluctuations secondary to vagal tone) [70]. The authors hypothesized that observations of increased cardiovascular events occurring during waking hours may be secondary to sudden increases in sympathetic activity. However, spectral analysis should not be the only form of variability analysis because there are patterns of variation that are present across the frequency spectrum, involving long-range organization and complexity.

## Power law

### Definition

Power law behaviour describes the dynamics of widely disparate phenomena, from earthquakes, solar flares and stock market fluctuations to avalanches. These dynamics are thought to arise from the system itself; indeed, the theory of self-organized criticality has been suggested to represent a universal organizing principle in biology [76]. It is illustrative to discuss the frequency distribution of earthquakes. A plot of the log of the power of earthquakes (i.e. the Richter scale) against the log of the frequency of their occurrence reveals a straight line with negative slope of -1. Thus, the probability of an earthquake may be determined for a given magnitude, occurring in a given region over a period of time, providing a measure of earthquake risk. In areas of increased earthquake activity, the line is shifted to the right, but the straight line relationship (and the slope) remains unchanged. Thus, the vertical distance between the straight line log–log frequency distributions or the intercept provides a measure of the difference in probabilities of an earthquake of all magnitudes between the two regions. Power law behaviour in physics, ecology, evolution, epidemics and neurobiology has also been described and reviewed [77].

Power laws describe dynamics that have a similar pattern at different scales, namely they are 'scale invariant'. As we shall see, detrended fluctuation analysis (DFA) is also a technique that characterizes the pattern of variation across multiple scales of measurement. A power law describes a time series with many small variations, and fewer and fewer larger variations; and the pattern of variation is statistically similar regardless of the size of the variation. Magnifying or shrinking the scale of the signal reveals the same relationship that defines the dynamics of the signal, analogous to the self-similarity seen in a multitude of spatial structures found in biology [78]. This scale invariant self-similar nature is a property of fractals, which are geometric structures pioneered and investigated by Benoit Mandelbrot [79]. Akin to a coastline, fractals represent structures that have no fixed length; their length increases with increased precision (magnification) of measurement, a property that confers a noninteger dimension to all fractals. In the case of a coastline, the fractal dimension lies between 1 (a perfectly straight coastline) and 2 (an infinitely irregular coastline). With respect to time series, the pattern of variation appears the same at different scales (i.e. magnification of the pattern reveals the same pattern) [78]. This is often referred to as fractal scaling. Of principal interest to clinicians and scientists is that one can measure the long range correlations that are present in a series of data and, as we shall see, measure the alterations present in states of illness.

### Calculation

As with frequency domain analysis (discussed above), the first step in the evaluation of the power law is the calculation of the power spectrum. This calculation, based on the fast Fourier transform (defined above), yields the frequency components of a series in time. By plotting a log–log representation of the power spectrum (log power versus log frequency), a straight line is obtained with a slope of approximately -1. As the frequency increases, the size of the variation drops by the same factor, and this patterns exists across many scales of frequency and variation, within a range consistent with system size and signal duration. Mathematically, power law behaviour is scale invariant; if a variable x is replaced by Ax', where A is a constant, then the fundamental power law relationship remains unaltered. A straight line is fitted using linear regression, and the slope and intercept are obtained (see Appendix 1).

### Interpretation and clinical implications

Power law behaviour has been observed for numerous physiological parameters and, relevant to clinicians, a change in intercept and slope is both present and prognostic in illness. Power law behaviour describes fluctuations in heart rate (first noted by Kobayashi and Musha [80]), foetal respiratory rate in lambs [81], movement of cells [82] and more. Power laws in pulmonary physiology were recently reviewed [83], noting a link between fractal temporal structure and fractal spatial anatomy. Alterations in the heart rate power law relationship (decreased or more negative slope) are present with ageing in healthy humans [84] as well as in patients with coronary artery disease [85]. Illness also confers changes in heart rate power law relationship. In over 700 patients with a recent myocardial infarction, as compared with age-matched control individuals, a steeper (more negative slope) power law slope was the best predictor of mortality evaluated [86]. In a random sample of 347 healthy individuals aged 65 years or older, a steep slope in the power law regression line (ß < -1.5) was the best univariate predictor of all-cause mortality, with an odds ratio for mortality at 10 years of 7.9 (95% confidence interval 3.7–17.0; *P *< 0.0001) [87]. Furthermore, only power law slope and a history of congestive heart failure were multivariate predictors of mortality in this cohort. Thus, changes in both slope and intercept have been documented to provide prognostic information in diverse patient populations.

Given that power law analysis is performed by plotting the log of spectral power versus the log of frequency using data derived from spectral analysis, what is the relationship between the two methods of characterizing variability? Although derived using the same data, the two methods assess different characteristics of signals. Spectral analysis measures the relative importance or contribution of specific frequencies to the underlying signal, whereas power law analysis attempts to determine the nature of correlations across the frequency spectrum. These analyses may have distinct and complementary clinical significance; for example, investigations of multiple HRV indices in patients following myocardial infarction [86] and in paediatric ICU patients [33] found that the slope of the power law had superior ability to predict mortality and organ failure, respectively, as compared with traditional spectral analysis.

### Limitation

Because determining power law behaviour requires spectral analysis, namely the determination of the frequency components of the underlying signal, the technique becomes problematic when applied to nonstationary signals. This limitation makes it difficult to draw conclusions regarding the mechanisms that underlie the alteration in dynamics observed in different patient groups. In addition, because power law behaviour measures the correlation between a large range of frequencies, it requires prolonged recording to achieve statistical validity. Nonetheless, as with the time and frequency domain analysis, valid clinical distinctions based on power law analysis have been demonstrated.

Specifically addressing the problem of nonstationarity, there is a problem in differentiating variations in a series of data that arise as an epiphenomenon of environmental stimuli (such as the effect of change in posture on heart rate dynamics) from variations that intrinsically arise from the dynamics of a complex nonlinear system [88,89]. Both lead to a nonstationary variations but nonetheless represent clinically distinct phenomena. The subsequent technique was developed to address this issue.

## Detrended fluctuation analysis

### Definition

Introduced by Peng and coworkers [90], DFA was developed specifically to distinguish between intrinsic fluctuations generated by complex systems and those caused by external or environmental stimuli acting on the system [88]. Variations that arise because of extrinsic stimuli are presumed to cause a local effect, whereas variations due to the intrinsic dynamics of the system are presumed to exhibit long-range correlation. DFA is a second measure of scale invariant behaviour because it evaluates trends of all sizes, trends that exhibit fractal properties (similar patterns of variation across multiple time scales). A component of the DFA calculation involves the subtraction of local trends (more likely related to external stimuli) in order to address the correlations that are caused by nonstationarity, and to help quantify the character of long-range fractal correlation representing the intrinsic nature of the system.

### Calculation

The calculation of DFA involves several steps (see Appendix 1). The analysis is performed on a time series, for example the intervals between consecutive heartbeats, with the total number of beats equal to N. First, the average value for all N values is calculated. Second, a new (integrated) series of data (also from 1 to N) is calculated by summing the differences between the average value and each individual value. This new series of values represents an evaluation of trends; for example, if the difference between individual NN intervals and the average NN interval remains positive (i.e. the interval between heartbeats is longer than the average interbeat interval), then the heartbeat is persistently slower than the mean, and the integrated series will increase. This trend series of data displays fractal, or scaling behaviour, and the following calculation is performed to quantify this behaviour. In this third step, the trend series is separated into equal boxes of length n, where n = N/(total number of boxes); and in each box the local trend is calculated (a linear representation of the trend function in that box using the least squares method). Fourth, the trend series is locally 'detrended' by subtracting the local trend in each box, and the root mean square of this integrated, detrended series is calculated, called F(n). Finally, it is possible to graph the relationship between F(n) and n. Scaling or fractal correlation is present if the data is linear on a graph of log F(n) versus log(n). The slope of the graph has been termed a, the scaling exponent. A single scaling exponent represents the limit as N and n approach infinity; however, applicable to real life data sets, the linear relationship between log F(n) and log n has been noted to be distinct for small n (n < 11) and large n (11 < n > 10,000), yielding two lines with two slopes, labelled the scaling exponents a_1 _and a_2_, respectively. For a more detailed description, see Appendix 1; excellent descriptions of the calculation of DFA may be found elsewhere [34,88].

### Interpretation and clinical applications

DFA offers clinicians the advantage of a means to investigate long range correlations within a biological signal due to the intrinsic properties of the system producing the signal, rather than external stimuli unrelated to the 'health' of the system. In addition, the calculation is based on the entire data set and is 'scale free', offering greater potential to distinguish biological signals based on scale specific measures [91]. Theoretically, the scaling exponent will vary from 0.5 (random numbers) to 1.5 (random walk), but physiological signals yield scaling exponents close to 1. A scaling exponent greater than 1.0 indicates a loss in long range scaling behaviour and a pathological alteration in the underlying system [88]. The technique was initially applied to detect long range correlations in DNA sequences [90] but has been increasingly applied to biological time signals.

As with other techniques of variability analysis, DFA has been used to evaluate cardiovascular variation. Elderly individuals [92], patients with heart disease [93] and asymptomatic relatives of patients with dilated cardiomyopathy who have enlarged left ventricles [94] all exhibit a loss of 'fractal scaling'. To date, a_1 _has demonstrated greater clinical discrimination of distinct heart rate data sets, as compared with a_2 _[88,94]. For example, a_1 _provided the best means of distinguishing patients with stable angina from age-matched control individuals; however, the correlation did not extend to angiographical severity of coronary artery disease [95]. In a retrospective evaluation of 2 hour ambulatory ECG recordings in the Framingham Heart Study [96], DFA was found to carry additional prognostic information that was not provided by traditional time and frequency domain measures. In a retrospective comparison between 24 hour HRV analysis using several techniques in patients post-myocardial infarction with or without inducible ventricular tachyarrhythmia [97], a decrease in the scaling exponent a_1 _was the strongest predictor of risk for ventricular arrhythmia. DFA was superior to spectral analysis in the analysis of HRV alteration in patients with sleep apnoea [98]. In a prospective, multicentre evaluation of HRV post-myocardial infarction, reduced short-term scaling exponent (a_1 _< 0.65) was the single best predictor of subsequent mortality [99]. In patients who had undergone coronary artery bypass surgery, reduced short-term scaling exponent in the postoperative period was the best predictor of a longer ICU stay, as compared with other HRV measures [100]. Thus, alteration in DFA scaling exponent (both increased and decreased) of heart rate fluctuation provides additional diagnostic and prognostic information that appears independent of time and frequency domain analysis.

In addition to cardiovascular variation, DFA has increasingly been applied to investigate other systems. Alterations in the scaling exponent of respiratory variation (inter-breath intervals) have been noted in elderly individuals [101]; and the finding of long-range correlations in breath–breath end-tidal carbon dioxide and oxygen fluctuations in healthy infants introduce novel avenues for investigation of respiratory illness [102]. Remarkably, the scaling properties of temperature measurements (every 10 min for 30 hours) are altered in association with ageing [103]. In addition, DFA provides meaningful information on EEG signals and has been utilized to distinguish normal individuals from stroke patients [104,105].

### Advantages and limitations

The principal advantage to DFA is the lack of confounding due to nonstationary data. DFA is readily calculated using a computer algorithm available through a cooperative academic internet resource, Physionet [106]. Although DFA represents a novel technological development in the science of variability analysis and has proven clinical significance, whether it offers information distinct from traditional spectral analysis is debated [107]. Data requirements are greater than with other techniques and have been suggested to include at least 8000 data points, as noted by empirical observations [88]. It is inappropriate to simply 'run' the DFA algorithm blindly on data sets; for example, a clear shift in the state of the cardiovascular system (e.g. spontaneous atrial fibrillation) would prohibit meaningful DFA interpretation. Finally, although appealing in order to simplify clinical comparison, the calculation of two scaling exponents (one for small and one for large n) represents a somewhat arbitrary manipulation of the results of the analysis. The assumption that the same scaling pattern is present throughout the signal remains flawed, and therefore techniques without this assumption are being developed and are referred to as multifractal analysis.

### Multifractal analysis

DFA is a monofractal technique, in that the assumption is that the same scaling property is present throughout the entire signal. Multifractal techniques provide multiple, possibly infinite exponents, such that the analysis produces a spectrum rather than a discrete value. For example, wavelet analysis is a multifractal analysis technique similar to DFA, which is capable of distinguishing the heart rate dynamics of patients with congestive heart failure from healthy control individuals [34]; a full discussion of multifractality of biological signals can be found elsewhere [108]. A separate technique recently introduced by Echeverría and colleagues [109] utilizes an a–ß filter (a technique imported from real-time radar tracking technology) to characterize heart rate fluctuations. Those authors suggested that this representation provides a superior means of identifying clinically distinct signals, and in order to demonstrate this they evaluated both theoretically and experimentally derived data sets. It remains unclear whether the added complexity and theoretical advantages of these techniques will afford consistent clinically significant improvements in the ability to distinguish physiological from pathological rhythms.

## Entropy analysis

### Definition

Entropy is a measure of disorder or randomness, as embodied in the Second Law of Thermodynamics, namely the entropy of a system tends toward a maximum. In other words, states tend to evolve from ordered statistically unlikely configurations to configurations that are less ordered and statistically more probable. For example, a smoke ring (ordered configuration) diffuses into the air (random configuration); the spontaneous reverse occurrence is statistically improbable to the point of impossibility. Entropy is the measure of disorder or randomness. Related to time series analysis, approximate entropy (ApEn) provides a measure of the degree of irregularity or randomness within a series of data. It is closely related to Kolmogorov entropy, which is a measure of the rate of generation of new information [110]. ApEn was pioneered by Pincus [111] as a measure of system complexity; smaller values indicate greater regularity, and greater values convey more disorder, randomness and system complexity.

### Calculation

In order to measure the degree of regularity of a series of data (of length N), the data series is evaluated for patterns that recur. This is performed by evaluating data sequences of length m, and determining the likelihood that other runs in the data set of the same length m are similar (within a specified tolerance r); thus two parameters, m and r, must be fixed to calculate ApEn. Once the frequency of occurrence of repetitive runs is calculated, a measure of their prevalence (negative average natural logarithm of the conditional probability) is found. ApEn then measures the difference between the logarithmic frequencies of similar runs of length m and runs with the length m+1. Small values of ApEn indicate regularity, given that the prevalence of repetitive patterns of length m and m+1 do not differ significantly and their difference is small. A derivation is included in Appendix 1, and a more comprehensive description of ApEn may be found elsewhere [112-114].

### Interpretation and clinical application

ApEn is representative of the rate of generation of new information within a biological signal because it provides a measure of the degree of irregularity or disorder within the signal. As such, it has been used as a measure of the underlying 'complexity' of the system producing the dynamics [111,112,115]. The clinical value of a measure of 'complexity' is potentially enormous because complexity appears to be lost in the presence of illness [114,116,117] (discussed in greater detail below).

As with other means of characterizing biological signals, ApEn has been most extensively studied in the evaluation of heart rate dynamics. Heart rate becomes more orderly with age and in men, exhibiting decreased ApEn [118]. Heart rate ApEn has demonstrated the capacity to predict atrial arrhythmias, including spontaneous [119] and postoperative atrial fibrillation after cardiac surgery [120], and to differentiate ventricular arrhythmias [121]. Heart rate ApEn is decreased in infants with aborted sudden infant death syndrome [122]; among adults, postoperative patients with ventricular dysfunction [123] and healthy individuals infused with endotoxin [124] exhibit reduced heart rate ApEn.

Because ApEn may be applied to short, noisy data sets, it was applied to assess the variation of parameters in which frequent sampling is more difficult (e.g. a blood test is necessary) and a paucity of data exists. This was most apparent in the evaluation of endocrine variability, as demonstrated in the following investigations. By applying ApEn to measurements of growth hormone (GH) every 5 min for 24 hours in healthy control individuals and patients with acromegaly, reduced orderliness (i.e. increased ApEn) was observed in acromegaly [125]; and normalization of GH ApEn values was demonstrated after pituitary surgery for acromegaly [126]. Increased disorderliness has been observed in insulin secretion in healthy elderly individuals as compared with young control individuals (insulin measured every minute for 150 min) [127], and in first-degree relatives of patients with non-insulin-dependent diabetes mellitus (insulin measured every minute for about 75 min) [128]. ApEn of adrenocorticotrophic hormone, GH, prolactin and cortisol levels (sampled every 10 min for 24 hours) is altered in patients with Cushing's disease [129,130]. Finally, altered dynamics of parathyroid hormone pulsatile secretion has been demonstrated in osteoperosis and hyperparathyroidism [131].

ApEn has also been used to evaluate neurological, respiratory and, recently, temperature variability. ApEn offers a means of assessing the depth of anaesthesia [132-134], and ApEn of tidal volume respiratory rate has been evaluated in patients with respiratory failure weaning from mechanical ventilation [135]. Alterations in respiratory variability are present in psychiatric illness; for example, increased entropy of respiration has been observed in patients with panic disorder [136]. Comparing chest wall movement and EEG activity in healthy individuals, sleep (stage IV) produced more regular breathing and more regular EEG activity [137]. Finally, demonstrating the remarkable potential and novel applications of variability analysis, ApEn of temperature measurements (every 10 min for 30 hours) revealed increased regularity and decreased complexity associated with age [103].

### Advantages and limitations

ApEn statistics may be calculated for relatively short series of data, a principal advantage in their application to biological signals. Referring to both theoretical analysis and clinical applications, Pincus and Golberger [112] concluded that m = 2 and r = 10–25% of the standard deviation of all the N values, and an N value of 10^m^, or preferably 30^m^, will yield statistically reliable and reproducible results (i.e. 100–900 data points). Pincus [114] also reported that ApEn is applicable to any system with at least 50 data points. In contrast to time domain measures of variability, which are independent of the sequence of the data set, ApEn required an evaluation of vectors representing consecutive data points, and thus the order of the data is integral to the calculation of ApEn and must be preserved during data harvest. Significant noise or nonstationary data compromise meaningful interpretation of ApEn [113]; therefore, it should not be used as the only means to measure signal characteristics.

### Sample and multiscale entropy

An inherent bias within the ApEn calculation exists because the algorithm counts similar sequences to a given sequence of length m, including counting the sequence itself (to avoid the natural logarithm of 0 within the calculations). As a result, ApEn can be sensitive to the size of the data set, giving inappropriately low values when the total number of data points is low; this, and a lack of consistency in differentiating signals when m and r are altered, have led to the development of a new family of statistics named sample entropy (SampEn), in which self-matches are excluded in the analysis [110]. SampEn has the advantage of being less dependent on the length of the data series in question, and has been applied to heart rate fluctuations in the paediatric ICU [138]. Finally, because both ApEn and SampEn are noted to evaluate differences between sequences of length m and m+1, they evaluate regularity on one scale only, the shortest one, and ignore other scales. Thus, given the temporal complexity of biological signals on multiple scales, a novel technique, multiscale entropy, was developed as a more robust measure of complexity [139]. Initial investigations of multiscale entropy have been promising [140], but comprehensive evaluation remains to be performed.

## Summary and discussion of variability techniques

The preceding sections highlight the considerable range of techniques that have been developed to characterize biological signals. Each with distinct theoretical background and significance, they contribute complementary information regarding signal characteristics. Time domain measures of variation represent an evaluation of overall, short-term or long-term variation, and are clinically proven as a means of identifying clinically significant alterations in biological signals, in particular with cardiovascular variability. Frequency domain analysis also has prognostic value, and has been useful in demonstrating the importance of sympathovagal balance in regulating HF and LF cardiovascular oscillations. Power law analysis contributes an analysis of fractal, long range correlations, allowing distinction between physiological and pathological signals with the slope and intercept of the power law. DFA also represents a means of detecting long range correlations, and is less bound by the stationarity assumption inherent to the other techniques. By measuring the degree to which sequences of data repeat themselves within a signal, ApEn provides a measure of signal irregularity, related to the rate of production of new information. Although techniques have shown consistent prognostic capacity, prediction of mortality is not the sole virtue of HRV analysis; separate techniques also may clarify mechanisms of disease [141]. Attempts to characterize biological signals should incorporate the 'toolkit' of techniques discussed in this review as well as the publication of raw data and code to facilitate comparison and development of this still young, exciting science [117].

### Interpretation and significance of altered variability

Following this review of the technology of variability analysis, the meaning of altered variability in biological signals must be addressed. A synthesis of the multiple but consistent theories regarding the significance of altered variability is presented to assist in the clinical application of this novel technology. A leading investigator within this field, Goldberger [142] proposed that increased regularity of signals represents a 'decomplexification' of illness, citing numerous examples of illness states with increased regularity of rhythms. For example, Cheyne–Stokes respiration, Parkinsonian gait, loss of EEG variability, preterminal cardiac oscillations, neutrophil count in chronic myelogenous leukaemia and fever in Hodgkin's disease all exhibit periodic, more regular variation in the dynamics of disease states [142]. Given that scale invariance is believed to be a central organizing principle of physiological structure and function, breakdown in this scale invariant, fractal behaviour, leads to uncorrelated randomness or more predictable behaviour, both representing a pathological alteration to the underlying system [78,84]. Thus, health is characterized by 'organized variability' and disease is defined by decomplexification, increased regularity and reduction in variability.

In contrast to the 'decomplexification' hypothesis, Vaillancourt and Newell [143] noted increased complexity and increased approximate entropy in several disease states, including acromegaly and Cushing's disease, and hypothesized that disease may manifest with increased or decreased complexity, depending on the underlying dimension of the intrinsic dynamic (e.g. oscillating versus fixed point). In a rebuttal, Goldberger [142] noted that increased complexity demonstrated by lower entropy (specifically ApEn) requires corroboration by other techniques, given potential problems with using ApEn as the only technique to assess variability. A rebuttal to the rebuttal (all published concurrently) [144] noted that others accept the fundamental premise that increased and decreased variability occur in disease.

In addition to the discussion regarding complexity, increased short-term variation in airway calibre in patients with asthma is observed, and reproduced experimentally with activation of airway smooth muscle with inhaled methacholine [5]. Given that smooth muscle activation is associated with increased metabolic rate, energy dissipation and an increased likelihood of statistically unlikely airway configurations, Macklem's hypothesis states that asthma is a disease of higher energy dissipation, greater distance from thermodynamic equilibrium, lower entropy and greater variation [5]. This suggests that health is defined by a certain distance from thermodynamic equilibrium; too close (decreased variation, too little energy dissipation, low entropy) or too far (increased variation and energy dissipation, high entropy) both represent pathological alterations.

The science of complex systems is intimately related to variability analysis. Taking a broad systems based interpretation, the human organism is a complex system or, more accurately, it is a complex system of complex systems. The host response to sepsis, shock, or trauma is an example of a biological complex system that is readily apparent to intensivists [3]. Every complex system has 'emergent' properties, which define its very nature and function, including the presence of health versus illness. Variability or patterns of change over time (in addition to connectivity or patterns of interconnection over space) represent technology with which to evaluate the emergent properties of a complex system, which may be physiological or pathological [3]. It is possible to conceive complex systemic host response in a phase space of variability parameters, in which health represents stable 'holes' in space, exhibiting marked systemic stability accompanied by specific patterns of variability (and connectivity). Illness represents an alteration from health, separate 'holes' with distinct patterns of variability. Often, it takes a major insult to change a stable healthy state to an illness state, which may have varying degrees of stability. It is within this complex systems conception of health and illness that the clinical utility of variability analysis may be appreciated.

### How can variability analysis improve outcome in the intensive care unit?

What does variability analysis offer that conventional monitoring does not? What is the clinical utility of this technology? We propose that multi-system continuous variability analysis offers the intensivist a unique monitoring tool that is capable of improving prognostication and directing therapeutic intervention. Intuitively, there is additional information in this analysis. Variability analysis tracks specific patterns of change in individual parameters over time (akin to calculating the first derivative or velocity in calculus). Monitoring patterns of change in variability continuously over time offers an additional dimension of analysis (akin to a second derivative evaluation or acceleration). Just as monitoring individual system variability offers an evaluation of the underlying individual system producing those dynamics, evaluating multisystem variability provides an evaluation of the whole, namely the systemic host response. By using variability analysis at different time points or, more powerfully, continuously over time, it is theoretically possible to track the 'system state' over time. Then, by selecting patients according to pathological patterns of variability and pursuing interventions with a therapeutic response or physiological alteration in variability, we hypothesize that outcomes in critically ill patients may be improved.

Why does this individualized variability directed therapy offer exciting clinical potential? First, as the host response is a complex system, response to intervention in individual patients is unpredictable, although response to an intervention may be statistically beneficial for a cohort of patients. Thus, only by evaluating the response to intervention in individual patients can it be ascertained that the intervention is beneficial in those patients. Interventions that have not proven beneficial for the 'average' patient may still be beneficial in selected individual patients, in whom pathological variability is both present and improved by therapy. In summary, continuous, individualized, variability directed, goal directed therapeutic intervention has numerous theoretical advantages over conventional epidemiological cohort analysis evaluating response to a single intervention given to a heterogeneous population of patients. This technology is well suited to the ICU, in which real-time, continuous, digital physiological data acquisition (including waveform analysis) has been demonstrated [145-147]. Unresolved questions include whether, how and when is it possible to convert pathological to physiological variability, to prod our patients from illness to health. Answering these questions will determine the impact variability analysis has on ICU patient outcome.

## Conclusion

The science of analyzing biological signals has undergone tremendous growth over the past decade, with the development of advanced computational methods that characterize the variation, oscillation, complexity and regularity of signals. These methods were developed in response to theoretical limitations of the others; however, all appear to have clinical significance. There is no consensus that any single technique is the single best means of characterizing and differentiating biological signals; rather, investigators agree that multiple techniques should be performed simultaneously to facilitate comparison between methods, techniques and studies. Variability analysis represents a novel means to evaluate and treat individual patients, suggesting a shift from epidemiological analytical investigation to continuous individualized variability analysis. Existing literature documents the clinical value of measuring variability to provide diagnostic, prognostic and pathophysiological information; future research must utilize this technology to improve care and the outcomes of our patients.

## Key messages

• 	A complex systems paradigm provides insights regarding research and treatment of critically ill patients.

• 	Variability analysis is the science of measuring the degree and character of patterns of variation of a time-series of a biologic parameter, in order to evaluate the state of the underlying complex system responsible for the biologic signal.

• 	Using techniques that measure overall variation, frequency contribution, scale-invariant variation and degree of disorder, altered variability in consistently present in illness states, and the degree of alteration provides a measure of prognosis.

• 	Using continuous multiogan variability analysis (CMVA), we hypothesize that goal-directed variability-directed therapeutic intervention will improve outcome and reduce mortality in critically ill patients, a novel individualized systems approach that complements analytical basic science and epidemiologic population science.

## Appendix 1: techniques of variability analysis

### Variability analysis

The description of means to characterize and differentiate biological signals, or sequences of data in time produced by biological systems, is referred to as 'variability analysis'. For example, a heart rate recording may be considered a series of intervals between consecutive heart beats, referred to as NN intervals (interval between consecutive normal sinus beats) or RR intervals (interval between consecutive R waves on an ECG). With the goal of providing a single means of characterizing a whole series of data, the following techniques were developed to perform variability analysis and applied to clinical data sets.

### Time domain analysis

Considered the simplest means of measuring variability, time domain analysis involves performing a statistical analysis of data expressed as a sequence in time. For example, SDNN (the standard deviation of NN intervals) has been used as a measure of HRV; greater variation yeilds higher standard deviation. Standard deviation is the square root of the average of the squared differences from the mean. SDANN (standard deviation of the average NN interval calculated over 5-min intervals within the entire period of recording) is a measure of longer term variation because the averaging process removes beat-to-beat variations. In contrast, the following variables were devised as a measure of short-term variation: RMSSD (square root of the mean squared differences of consecutive NN intervals), NN50 (number of pairs of adjacent NN intervals differing by more than 50 ms), and pNN50 (proportion of NN intervals differing by more than 50 ms = NN50 divided by total number of NN intervals).

### Frequency domain analysis

Physiological data collected as a series in time may be considered a sum of rhythmic oscillations with distinct frequencies. Conversion from time domain to frequency domain analysis is performed most commonly using the Fourier transform, which decomposes the signal into a series of sine and cosine waves with frequencies that are multiples of the fundamental frequency (reciprocal of the time length to the input data record); the fast Fourier transform is a discrete Fourier transform that reduces the number of computations. The result of the Fourier transform is a complex number (a number multiplied by the square root of -1) for each frequency, the square of which is considered the spectral power of that frequency. The whole process is called spectral analysis, because it provides an evaluation of the spectral power (amplitude) of the contributing frequencies of an underlying signal.

### Power law analysis

Power law behaviour may be described by the following equation:

F(x) = ax^ß^

Where a and ß are constants. Taking the logarithm of both sides, a straight line (graph log f [x] versus log x) with slope ß and intercept log a is revealed:

Log f(x) = log (ax^ß^) = log a + log x^ß ^= log a + ß log x

Thus, power law behaviour is scale invariant; if a variable x is replaced by Ax', where A is a constant, then the fundamental power law relationship remains unaltered. If dynamics follow a power law, a log–log representation of the power spectrum (log power versus log frequency) reveals a straight line, always within a defined range consistent with the size and duration of the system. The straight line is fitted using linear regression, and the slope ß and intercept can readily be obtained. When ß = -1, the dynamics are described as 1/f noise. Power law behaviour describes the dynamics of widely disparate phenomena, including heart rate fluctuations, inter-breath intervals, earthquakes, solar flares, stock market fluctuations, and avalanches.

### Detrended fluctuation analysis

Variations that arise because of extrinsic stimuli are presumed to cause a local effect, whereas variations due to the intrinsic dynamics of the system are presumed to exhibit long range correlation. DFA attempts to quantify the presence or absence of long range scale-invariant (fractal) correlation.

The first step in the technique to calculate DFA is to map a biological signal, such as a series of heart beats, to an integrated series. The integrated series is calculated by the sum of the differences between individual inter-beat intervals represented as NN_i _and the average interbeat interval for the whole data set, equal to NN_ave_.

y(k) = S_i = 1_^N ^(NN_i _- NN_ave_)

This series y(k) represents an evaluation of trends; for example, if the difference NN_i _- NN_ave _remains negative (heart beat is persistently faster than the mean), then y(k) increases as k increases. This trend function y(k) is then separated into equal boxes of length n, where n = N/(total number of boxes). In each box, the local trend y_n_(k) is calculated as a linear representation of the function y(k) in that box using the least squares method. Least squares analysis involves the principle of optimization of the estimate based on minimizing the sum of the squared differences from the values predicted by the model. The series y(k) is then 'detrended' by subtracting the local trend y_n_(k). The root mean square of this integrated and detrended series is represented by the following:

F(n) = v (1/N S_k = 1_^N ^[y(k)^2 ^- y_n_(k)^2^])

By performing this analysis for all values of n, it is possible to calculate the relationship between F(n) and n. Scaling or fractal correlation is present if the data is linear on a graph of log F(n) versus log(n). The slope of the graph has been termed a, the scaling exponent, which will vary from 0.5 (white noise or uncorrelated random data) to 1.5 (Brownian noise or integrated white noise or random walk). When a = 1, behaviour corresponds to the 1/f noise. As a increases above 1 to 1.5, behaviour is no longer determined by a power law. Because the linear relationship between log F(n) and log(n) appears to have two distinct linear segments, one for small (n < 11) and large n (n > 11), the slopes of both lines are calculated separately and termed a_1 _and a_2_, respectively; repeatedly, a_1 _has proven superior to a_2 _in terms of prognostic ability.

### Approximate entropy

ApEn is a measure of 'irregularity'; smaller values indicate a greater chance that a set of data will be followed by similar data (regularity), and a greater value for ApEn signifies a lesser chance of similar data being repeated (irregularity). To calculate ApEn of a series of data, the data series is evaluated for patterns that recur. This is performed by evaluating data sequences or runs of length m, and determining the likelihood that other runs of length m are similar, within a tolerance r. Thus, two parameters, m and r, must be fixed to calculate ApEn. Increased regularity is associated with illness.

The following is a description of the calculation of ApEn. Given any sequence of data points u(i) from i = 1 to N, it is possible to define vector sequences x(i), which consist of length m and are made up of consecutive u(i), specifically defined by the following:

x(i) = (u [i], u [i + 1], ... u [i + m - 1])

In order to estimate the frequency that vectors x(i) repeat themselves throughout the data set within a tolerance r, the distance d(x [i],x [j]) is defined as the maximum difference between the scalar components x(i) and x(j). Explicitly, two vectors x(i) and x(j) are 'similar' within the tolerance or filter r (i.e. d(x [i],x [j]) = r) if the difference between any two values for u(i) and u(j) within runs of length m are less than r (i.e. |u(i + k) - u(j+k)| = r for 0 = k = m). Subsequently, C_i_^m^(r) is defined as the frequency of occurrence of similar runs m within the tolerance r:

C_i_^m^(r) = (number of j such that d(x [i],x [j]) = r)/(N - m - 1), where j = (N - m - 1)

Taking the natural logarithm of C_i_^m^(r), F^m^(r) is defined as the average of ln C_i_^m^(r):

F^m^(r) = S_i _ln C_i_^m^(r)/(N - m - 1), where S_i _is a sum from I = 1 to (N - m - 1)

F^m^(r) is a measure of the prevalence of repetitive patterns of length m within the filter r.

Finally, approximate entropy, or ApEn(m,r,N), is defined as the natural logarithm of the relative prevalence of repetitive patterns of length m as compared with those of length m + 1:

ApEn(m,r,N) = F^m^(r) - F^m+1^(r)

Thus, ApEn(m,r,N) measures the logarithmic frequency that similar runs (within the filter r) of length m also remain similar when the length of the run is increased by 1. Thus, small values of ApEn indicate regularity, given that increasing run length m by 1 does not decrease the value of F^m^(r) significantly (i.e. regularity connotes that F^m ^[r] ~ F^m+1 ^[r]). ApEn(m,r,N) is expressed as a difference, but in essence it represents a ratio; note that F^m^(r) is a logarithm of the averaged C_i_^m^(r), and the ratio of logarithms is equivalent to their difference.

## Competing interests

None declared.

## Abbreviations

ApEn = approximate entropy; DFA = detrended fluctuation analysis; EEG = electroencephalogram; GH = growth hormone; HF = high frequency; HRV = heart rate variability; ICU = intensive care unit; LF = low frequency; NN50 = number of pairs of adjacent NN intervals differing by more than 50 ms; pNN50 = proportion of NN intervals differing by more than 50 ms; RMSSD = square root of the mean squared differences of consecutive NN intervals; SampEn = sample entropy; SDANN = standard deviation of the average NN interval calculated over 5 min intervals within the entire period of recording; SDNN = standard deviation of a series of NN intervals; ULF = ultralow frequency; VLF = very low frequency.

## Figures and Tables

**Table 1:** Techniques to characterize variability

Variability analysis	Description	Advantages	Limitations	Output variables
Time domain	Statistical calculations of consecutive intervals	Simple, easy to calculate; proven clinically useful; gross distinction of high and low frequency variations	Sensitive to artifact; requires stationarity; fails to discriminate distinct signals	SD, RMSDD Specific to HRV: SDANN, pNNx
	Frequency distribution (plot number of observations falling in selected ranges or bins)	Visual representation of data; can fit to normal or log-normal distribution	Lacks widespread clinical application; arbitrary number of bins	Skewness (measures symmetry): positive (right tail) versus negative (left) Kurtosis (measures peakedness): flatter top (<0) versus peaked (>0)
Frequency domain	Frequency spectrum representation (spectral analysis)	Visual and quantitative representation of frequency contribution to waveform; useful to evaluate relationship to mechanisms; widespread HRV evaluation	Requires stationarity and periodicity for validity; sensitive to artifact; altered by posture, sleep, activity	Total power (area under curve) Specific to HRV: ULF (<0.003 Hz), VLF (0.003–0.04 Hz), LF (0.04–0.15 Hz), HF (0.15–0.4 Hz) Time spectrum analysis
Scale invariant (fractal) analysis	Power law: log power versus log frequency	Ubiquitous biologic application; characterization of signal with single linear relationship; enables prognostication	Requires stationarity and periodicity; requires large datasets	Slope of power law Intercept of power law
	DFA	Identifies intrinsic variations 2°system (versus external stimuli), does not require stationarity	Requires large datasets (>8000 patients)	Scaling exponent a_1 _(n < 11) Scaling exponent a_2 _(n > 11) a–ß filter
Entropy	Measures the degree of disorder (information or complexity)	Unique representation of data; requires fewest data points (100–900 patients)	Needs to be complemented by other techniques	ApEN SampEN Multi-scale entropy

ApEn, approximate entropy; DFA, detrended fluctuation analysis; HF, high frequency; HRV, heart rate variability; LF, low frequency; pNNx, proportion greater than x ms; RMSDD, root mean square of standard deviation; SampEn, sample entropy; SD, standard deviation; SDANN, standard deviation of 5 min averages; ULF, ultralow frequency; VLF, very low frequency.

**Table 2:** Evidence for altered patterns of variability in illness states

Variability analysis	Cardiac	Respiratory	Neurological	Miscellaneous	Critical care
Time domain	?HRV ??mortality risk in elderly, CAD, post-MI, CHF and dilated cardiomyopathy [14–24]	Altered frequency distribution of airway impedance in asthma [5] Altered respiratory variability (?kurtosis) in sleep apnoea [148]			
Frequency domain	Altered spectral HRV analysis?illness severity in cardiac disease (CHF [50–52], hypertension [53,54], CAD [55,56], angina [57], MI [58]) and noncardiac disease (hypovolaemia [49], chronic renal failure [59], diabetes mellitus [60], anaesthesia [61])				?Total HRV, ?LF and ?LF/HF HRV following trauma [149], sepsis and septic shock in the ICU [62,64,68,150,151] and in ER patients [63]
Power law analysis	Altered HRV power law (?HRV left shift and steeper slope) with age [84], CAD [85] and post-MI [86]	?Respiratory variability (right shift) in patients with asthma [7] ?Variability of foetal breathing with maternal alcohol intake [152]	Altered variability in gait analysis [153–155] and postural control [156] with ageing and neurological disease Altered variability of mood?psychiatric illness [157–159]	Haematological: altered leucocyte dynamics [160,161] observed in haematological disorders (e.g. cyclic neutropenia)	Altered HRV power law (?HRV left shift)??mortality risk in paediatric ICU patients [33]
DFA	Altered DFA scaling exponent?age [92], heart disease [93–96], post-ACBP [100], prearrhythmias [97], patients with sleep apnoea [98], and ?mortality risk post-MI [99]	Altered respiratory variability (?DFA scaling exponent)?age[101]		Temperature: altered temperature measurements?age[103]	?Heart rate DFA scaling exponent?septic shock[162] and procedures[61] in paediatric ICU patients
Entropy	?HR ApEn?age [118], ventricular dysfunction [123], occurs prior to arrhythmias [119–121]	Greater respiratory irregularity in patients with panic disorder [136]	Altered EEG entropy with anaesthesia[132,163,164]	Endocrine: ?ApEn of GH [125,126], insulin [127,128], ACTH, GH, PRL [129,130], PTH [131]?age and/or illness	?HR ApEn?healthy individuals infused with endotoxin [124] ?TV ApEn in respiratory failure [135]

?, decreased; ?, increased; ?, is associated with; ACBP, aorto–coronary bypass procedure; ACTH, adrenocorticotrophic hormone; ApEn, approximate entropy; CAD, coronary artery disease; CHF, congestive heart failure; DFA, detrended fluctuation analysis; EEG, electroencephalogram; ER, emergency room; GH, growth hormone; HF, high frequency; HRV, heart rate variability; ICU, intensive care unit; LF, low frequency; MI, myocardial infarction; PRL, prolactin; PTH, parathyroid hormone; TV, tidal volume.
